# Signaling pathways and mesenchymal transition in pediatric high-grade glioma

**DOI:** 10.1007/s00018-017-2714-7

**Published:** 2017-11-21

**Authors:** Michaël H. Meel, Sophie A. Schaper, Gertjan J. L. Kaspers, Esther Hulleman

**Affiliations:** 1 Departments of Pediatric Oncology/Hematology, Neuro-oncology Research Group, Cancer Center Amsterdam, VU University Medical Center, De Boelelaan 1117, 1081HV Amsterdam, The Netherlands; 2 Princess Máxima Center for Pediatric Oncology, Uppsalalaan 8, 3584CT Utrecht, The Netherlands

**Keywords:** High-grade glioma, Diffuse intrinsic pontine glioma, Epithelial-to-mesenchymal transition, Glioblastoma, Chemoresistance, Radioresistance

## Abstract

Pediatric high-grade gliomas (pHGG), including diffuse intrinsic pontine gliomas (DIPG), are the most lethal types of cancer in children. In recent years, it has become evident that these tumors are driven by epigenetic events, mainly mutations involving genes encoding Histone 3, setting them apart from their adult counterparts. These tumors are exceptionally resistant to chemotherapy and respond only temporarily to radiotherapy. Moreover, their delicate location and diffuse growth pattern make complete surgical resection impossible. In many other forms of cancer, chemo- and radioresistance, in combination with a diffuse, invasive phenotype, are associated with a transcriptional program termed the epithelial-to-mesenchymal transition (EMT). Activation of this program allows cancer cells to survive individually, invade surrounding tissues and metastasize. It also enables them to survive exposure to cytotoxic therapy, including chemotherapeutic drugs and radiation. We here suggest that EMT plays an important, yet poorly understood role in the biology and therapy resistance of pHGG and DIPG. This review summarizes the current knowledge on the major signal transduction pathways and transcription factors involved in the epithelial-to-mesenchymal transition in cancer in general and in pediatric HGG and DIPG in particular. Despite the fact that the mesenchymal transition has not yet been specifically studied in pHGG and DIPG, activation of pathways and high levels of transcription factors involved in EMT have been described. We conclude that the mesenchymal transition is likely to be an important element of the biology of pHGG and DIPG and warrants further investigation for the development of novel therapeutics.

## Introduction

Brain tumor is the number one cause of cancer-related morbidity and mortality in children and adolescents. Among pediatric brain tumors, high-grade gliomas (HGG), and especially, diffuse intrinsic pontine gliomas (DIPG) carry the worst prognosis with a 5-year survival of less than 10% [1]. For DIPG, median survival is only 11 months [2]. On a molecular level, DIPG is characterized by a high prevalence of mutations in genes encoding Histone 3 (mainly *H3F3A* and *HIST1H3B*), leading to a lysine-to-methionine substitution at position 27 in the N-terminal tail of the histone protein, causing global H3K27 hypomethylation. Supratentorial pHGG often carry a G34R/V mutation in *H3F3A*, resulting in decreased H3K36 methylation [3–7]. These mutations set the pediatric brain tumors apart from their adult counterparts, and emphasize the necessity to research them independently. Given the highly infiltrative nature and delicate location of DIPG in the brainstem, surgery is generally impossible. Historically, the diagnosis of DIPG was based on MRI appearance of the tumor, and surgical biopsies were rarely performed. However, in recent years, many institutes are re-introducing biopsies in early clinical phase studies to enable molecular characterization of the tumor [8]. In contrast, supratentorial pHGG is almost always subjected to surgery. Although rarely resulting in a cure of the patient, the extent of surgical resection is one of the most important independent clinical prognostic factors [9]. Additionally, pediatric HGG and DIPG are extremely chemoresistant, partially due to the blood–brain barrier (BBB) and partially due to intrinsic factors [10]. To this day, no chemotherapy regimen is known to produce any response in DIPG at acceptable toxicity levels, and only a very modest survival benefit of chemotherapy is seen in supratentorial pediatric HGG [9]. The only treatment that definitively benefits patients with pHGG and DIPG is intensive radiotherapy, which alleviates symptoms and slightly prolongs survival, but offers no chance for a cure. In adult glioblastoma (GBM), as in other types of cancer, the intrinsic chemoresistance has been related to the process of epithelial-to-mesenchymal transition (EMT), a transcriptional process in which epithelial cells adopt mesenchymal properties by loss of cell–cell adhesion, acquisition of migratory and invasive properties and loss of cell polarity [11, 12]. In tumors, in general, EMT contributes to initiation, invasion, metastasis to distant sites and resistance to chemo- and/or radiotherapy. Additionally, it is believed to stimulate the development and maintenance of a cancer stem cell (CSC) population within the tumor [13–15]. We hypothesize that EMT plays an equally important role in pediatric HGG and DIPG, by which it would be responsible for the intrinsic chemoresistance and diffuse nature of these tumors.

EMT is induced by external factors, such as cytokines, growth factors and hypoxia. Different signaling pathways lead to activation of EMT transcription factors; most importantly SNAI1 (SNAIL), SNAI2 (SLUG), ZEB1, ZEB2 and TWIST. These transcription factors inhibit the expression of genes that promote cell–cell contact, most importantly E-cadherin, and induce genes that promote migration and stemness [11, 16–18]. This review serves to summarize current knowledge on the process of mesenchymal transition in pediatric HGG and DIPG.

## Transcription factors involved in the mesenchymal transition

### SNAIL family of transcription factors

**Fig. 1:**
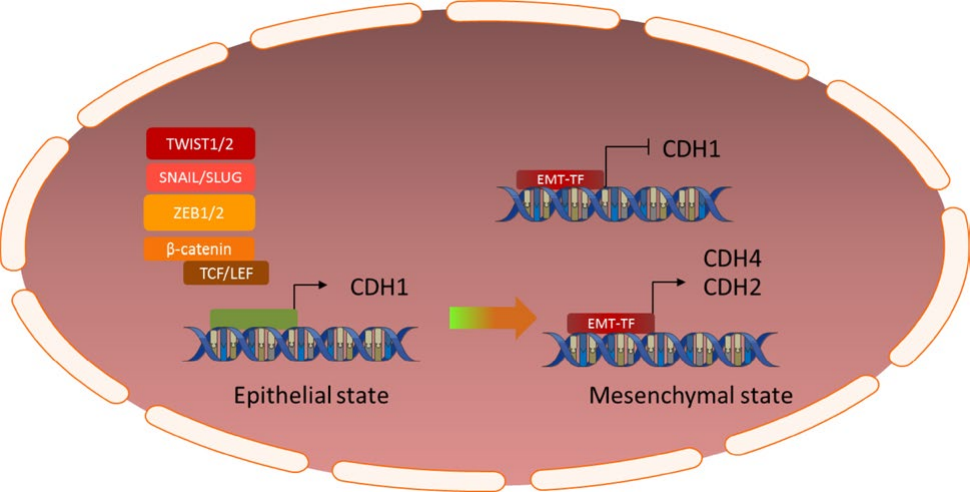
Graphic illustration of the cadherin switch; EMT transcription factors inhibit expression of E-Cadherin (CDH1) and induce expression of N- and/or R-Cadherin, a crucial event in the (epithelial-to-)mesenchymal transition

One of the most important regulators of the mesenchymal transition is the SNAIL family of master transcriptional regulators, consisting of three members; SNAIL (SNAI1), SLUG (SNAI2) and SMUC (SNAI3). SNAIL family transcription factors are basic zinc finger transcription factors with a broad variety of functions during normal development and cancer progression [19–22]. Most research has been dedicated to unraveling the role of these factors in the epithelial-to-mesenchymal transition. Central to this role is their ability to repress E-cadherin transcription through direct binding to the *CDH1* promoter and concurrently increase the expression of other cadherins, such as N-cadherin (CDH2) [11, 23]. Furthermore, SNAIL transcription factors have a profound impact on epigenetic regulation of transcription through recruitment of several histone methyltransferases, demethylases, acetyltransferases and deacetylases to the histone-DNA complex, thus resulting in the transcription of genes associated with a mesenchymal phenotype and concurrent repression of epithelial genes, such as occludins, claudins, mucins and cytokeratins [24, 25] (Fig. 1).

Among the most important mesenchymal proteins are vimentin, fibronectin and matrix metalloproteinases [19]. These proteins are involved in the structural integrity of mesenchymal cells, both through integrin signaling and their capacity to modify the extracellular environment [26, 27]. In cancer progression, this expression of mesenchymal genes [28] allows cancer cells to become less dependent of cell–cell interactions, enabling them to migrate, invade surrounding tissues and eventually metastasize [20, 29–32]. Additionally, SNAIL upregulates the expression of *ZEB1*, another master transcriptional regulator of the mesenchymal transition [33]. Finally, an important role for these transcription factors lies in their ability to render cancer cells resistant to therapy via incompletely understood mechanisms, as is the case for example in cisplatin-resistant NSCLC, and especially relevant for the current review, GBM [12, 19, 20]. Importantly, SNAIL is found to be a key player in mesenchymal transition in malignant gliomas, including DIPG, increasing invasion migration, focal adhesion and proliferation [34–38]. SNAIL levels increase after irradiation [34] and its expression is correlated with tumor grade and survival [39]. Although SLUG is not as extensively studied in gliomas as SNAIL, it too is correlated with invasion, histological grade and glioblastoma growth [40].

### ZEB family of transcription factors

Another group of master regulators of the mesenchymal transition is the ZEB family of transcription factors, comprising ZEB1 and ZEB2. These transcription factors contain two zinc finger domains which allow DNA binding at E-boxes within the promoter regions of target genes such as E-cadherin and N-cadherin [11]. Whether ZEB1 and ZEB2 function as transcriptional activators or repressors is determined by their interaction with cofactors, such as the Smad family of signal transduction proteins, and other transcription factors. The net result of ZEB activity, however, is always a skewing of gene expression to a more mesenchymal phenotype [41]. Overexpression of ZEB1 and ZEB2 is a common phenomenon in many types of cancer, where it is associated with the maintenance of a cancer stem cell phenotype as well as metastasis and therapy resistance, such as paclitaxel resistance in breast cancer cells [42–44].

ZEB1 has been found to be correlated to a highly infiltrative tumor phenotype in both adult and pediatric high-grade glioma, partially in relation to hypoxia [45, 46]. Moreover, ZEB1 expression is linked to poor temozolomide response and a shorter survival of GBM patients [45]. Interestingly, ZEB1 has been shown to be important for EMT in HGG driven by PDGFRα activation [47]. As pediatric HGG commonly possesses PDGFRα overexpression and activation, this ZEB1-PDGFRα axis may well be important for the mesenchymal transition of pHGG [38, 48–50]. Although less well studied, ZEB2 expression also appears to be correlated with tumor grade and mesenchymal properties in high-grade gliomas [51]. Specific data on the role of ZEB2 in pediatric HGG and DIPG is lacking.

### TWIST family of transcription factors

A third group of master regulators of the mesenchymal transition is the TWIST family of basic helix-loop-helix (bHLH) transcription factors, of which TWIST1 and TWIST2 have been studied most. Basic HLH transcription factors, such as the zinc finger transcription factors, exert their action through binding to the E-boxes in the promotor regions of target genes. In this manner, as with the SNAIL and ZEB families of transcription factors, TWIST represses E-cadherin and enhances N-cadherin expression, thereby promoting tumor cell migration, invasion, metastasis and chemoresistance in a broad variety of cancer types [52–55].

TWIST1 has been repeatedly investigated in adult glioma, in which its expression is correlated with higher tumor grade and poorer survival of patients. Mechanistically, this is attributed to the increased migratory and invasive capacities of glioma cells by TWIST1 activity [56]. Additionally, TWIST1 expression has been associated with resistance of glioblastoma cells to temozolomide [57]. Importantly, one study found that a reduction of TWIST1 expression in glioma stem cells, achieved by co-culturing these stem cells with mesenchymal stem cells, induces a mesenchymal-to-epithelial transition (MET), the inverse process of EMT, in these cells [58]. Specific data on the role of TWIST in pediatric HGG and DIPG is not available.

### SIX family of transcription factors

Less well studied, but nonetheless relevant, is the SIX family of homeobox transcription factors, of which only SIX1 has received considerable attention. These transcription factors are highly expressed in embryogenesis, where they have an important role in embryonal progenitor cell proliferation and survival. When SIX proteins are aberrantly expressed in non-embryonal tissue they can function as oncogenes, promoting cell proliferation and metastasis in various cancer types, partially through activation of the TGF-β and RAS/MAPK pathways [59–63].

SIX1 has mostly been studied in breast cancer, where it correlates with worse patient survival and induces EMT in cancer stem cells, thereby causing malignant progression [61, 64]. Furthermore, SIX1 has been shown to play a role in EMT in high-grade glioma cells, resulting in increased cell invasiveness [65, 66]. To date, no research has addressed the role of the SIX transcription factors in pediatric HGG or DIPG.

## External initiation of the epithelial-to-mesenchymal transition

**Fig. 2:**
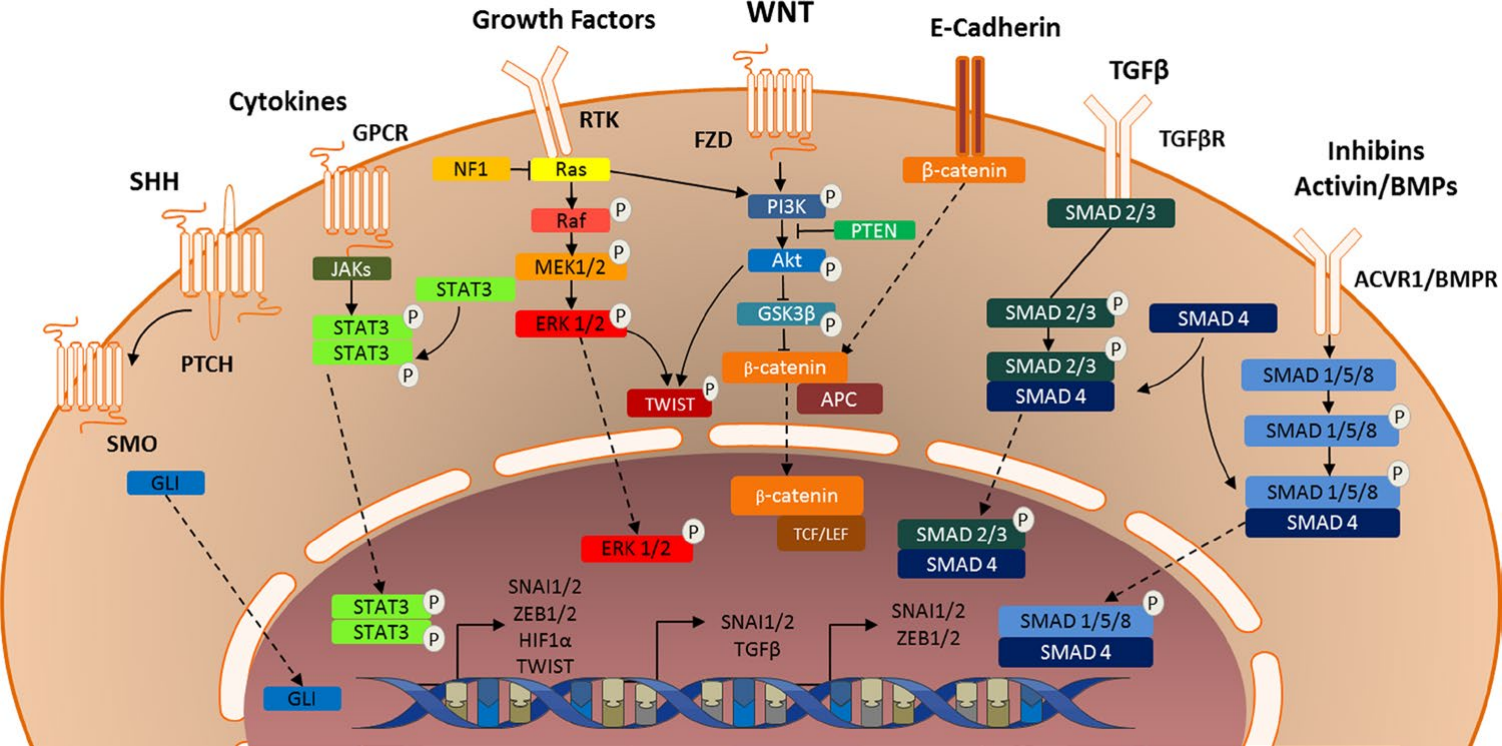
Graphic illustration of signaling pathways conveying external signals to transcription of mesenchymal genes, thereby initiating and maintaining the (epithelial-to-)mesenchymal transition

The epithelial-to-mesenchymal transition is commonly initiated by external stimuli, such as growth factors, cytokines and/or hypoxia. These external stimuli trigger intracellular signaling cascades that ultimately result in an upregulation of transcriptional regulators, such as SNAIL and ZEB [11]. Key pathways in this process include TGFβ and activin signaling, WNT signaling, JAK/STAT, RTK and SHH signaling (Fig. 2).

### TGF-β and activin signaling

One of the first mechanisms discovered by which external triggers could induce EMT is the TGF-β signaling pathway. The TGF-β superfamily consists of two activins, three TGF-βs and many bone morphogenetic proteins that all function through homo- or heterodimers of receptors. Binding of TGF-β or related proteins to their receptors causes phosphorylation of tyrosine, serine and/or threonine residues in the cytoplasmic domain of the receptor. Via a series of intermediate events, this results in phosphorylation of SMAD2 and SMAD3, which in turn form a complex with and phosphorylate SMAD4 [11, 67] (Fig. 2). This trimeric complex translocates to the nucleus, where it both causes expression of and interacts with transcription factors capable of inducing EMT, such as SNAIL, SLUG, ZEB1 and ZEB2 [11]. Furthermore, activation of TGF-β receptors by their ligands can activate canonical signaling pathways such as the PI_3_K/AKT, MAPK and JNK pathways, which will be discussed later on [68, 69].

In pediatric high-grade glioma, a special role seems to be reserved for the activins within the TGF-β superfamily of proteins. Recent studies have identified activating mutations gene encoding *Activin Receptor Type 1 (ACVR1)* in a subset of diffuse midline gliomas [50, 70, 71]. Mutations in this gene are known to cause ligand-independent receptor activation with subsequent phosphorylation and nuclear translocation of SMAD1, SMAD 5 and SMAD8 [70, 72–74]. There, they too form a complex with SMAD4 to function as a transcription factor regulating expression of genes inducing EMT [74]. As with many proteins involved in EMT, ACVR1 seems to be mainly involved in embryogenesis under normal circumstances [73]. This observation raises the possibility that ACVR1 mutations induce the mesenchymal transition in diffuse midline gliomas. This hypothesis is supported by the observation that ACVR1 mutations are associated with a mesenchymal gene expression profile in diffuse intrinsic pontine glioma [38]. This makes both ACVR1 and its downstream effectors interesting targets for the treatment of diffuse intrinsic pontine gliomas, especially when combined with other therapeutic modalities.

### WNT signaling in the mesenchymal transition

In the canonical WNT/β-catenin pathway, WNT first binds to Frizzled cell surface receptors and low-density lipoprotein-related receptor proteins 5 or 6 [75–78]. The activation of Frizzled receptors results in phosphorylation of AKT, followed by inhibitory phosphorylation of GSK3β, resulting in stabilization of SNAIL protein [11]. Moreover, GSK3β inhibition enables β-catenin to act as a transcription factor. Normally, GSK3β is part of a complex with the proteins APC and AXIN, the so-called destruction complex. This complex phosphorylates and thereby degrades β-catenin [79, 80]. Therefore, when GSK3β is inhibited, β-catenin accumulates in the cytoplasma and translocates to the nucleus [81, 82]. There, it serves as a subunit of a high-mobility group (HMG) box transcription factor complex. In association with TCF/LEF, β-catenin induces the expression of genes encoding EMT transcription factors [83, 84]. In addition, β-catenin is an important part of the E-cadherin/β-catenin complex that regulates cellular adhesion and migration, making the WNT/β-catenin pathway a key mechanism regulating EMT in cancer (stem) cells [13].

The WNT/β-catenin pathway has many interactions with other pathways involved in EMT. For example, it cooperates with TGF-β signaling to induce EMT in palate medial-edge epithelial cells [11, 85] and increases HIF-1α expression in prostate and liver cancer, which in turn results in EMT [86–90]. In glioma there is ample evidence for the relevance of the WNT/β-catenin pathway in the mesenchymal transition. First, expression of pathway components correlates to survival in glioma patients [91–93]. Second, WNT/β-catenin signaling has been shown to promote glioma cell proliferation, migration, invasion and radio- and chemoresistance [94–104]. Most importantly, however, EMT-mediated chemoresistance can be reversed in glioblastoma stem cells by treatment with secreted Frizzled-related protein (sFRP4), which functions as a WNT antagonist [105, 106]. A recent study demonstrated that the role of WNT/β-catenin signaling in pediatric HGG, in contrast to adult GBM, is very limited [107].

Besides the canonical WNT/β-catenin pathway, two non-canonical WNT signaling pathways have been identified; the planar cell polarity (PCP) and WNT/Ca^2+^ pathways [108, 109]. Although not extensively studied, components of these pathways, most importantly WNT5A, have been shown to be involved in the induction of EMT in several types of cancer [110–114]. Both non-canonical WNT pathways have been shown to play a role in adult glioma, where they increase stemness and invasive properties of glioma cells [109, 115–122]. Data on the role of non-canonical WNT signaling in pHGG and DIPG has not been published to this day.

### PI_3_K/AKT signaling in the mesenchymal transition

Activation of well-known receptor tyrosine kinases (RTK), such as the EGF, FGF and PDGF receptors, as well as G-protein coupled receptors (GPCR), is known to induce EMT via activation of the canonical PI_3_K/AKT and RAS/MAPK pathways. The role of the PI_3_K/AKT pathway in EMT is well established; after activation of the pathway, AKT inhibits GSK3β by phosphorylating serine 9. This in turn prevents GSK3β from phosphorylating SNAIL or SLUG, thus inhibiting their ubiquitination and subsequent proteasomal degradation [123, 124]. Via this mechanism, activation of the PI_3_K/AKT pathway causes stabilization of SNAIL family proteins, thereby inducing EMT [11].

In addition, activation of the PI_3_K/AKT pathway induces the transcription of ZEB1 by reducing GSK3β-mediated phosphorylation of β-catenin, thereby stabilizing the protein and inducing *ZEB1* transcription via a β-catenin/TCF4 complex [125, 126]. Finally, AKT-mediated TWIST1 phosphorylation promotes EMT, as for example in the case of breast cancer metastasis, by modulating its transcriptional target TGF-β2, leading to enhanced TGF-β receptor signaling. This signaling pathway in turn maintains hyperactive phosphoinositide 3-kinase (PI_3_K)/AKT signaling), resulting in induction of EMT [127]. The PI_3_K/AKT pathway, which is commonly activated in malignant cells, has been shown to induce EMT in several tumor types. In certain types of cancer, inhibition of the overactive PI_3_K/AKT signaling resulted in a decrease of mesenchymal characteristics and (re-)sensitization of cancer cells to chemo- and radiotherapy [128–131].

There are several EMT pathways that cross talk with the PI_3_K/AKT/GSK3β pathway in glioma, for example the HIF-1α and the WNT/β-catenin pathways [132–135]. Moreover, loss of PTEN is often found in adult GBM, leading to aberrant activation of the PI_3_K pathway [136]. In line with this finding, PI_3_K/mTOR kinase inhibitors provide a promising glioma therapy, as those drugs sensitize tumor cells to radiation and chemotherapy [137]. In pediatric HGG and DIPG, activating mutations in genes encoding PI_3_K are found in 15–26% of patients [138–140], which may contribute to the mesenchymal transition in these tumors.

### RAS/MAPK signaling in the mesenchymal transition

Activation of RTKs also leads to signaling via the RAS/MAPK pathway [141]. Within this cascade, ERK1/2 phosphorylation and subsequent nuclear translocation leads to an increase in the transcription of *SNAIL* and *SLUG* and regulators of cell invasion and motility through activation of RHO-GTPases [11, 142]. Additionally, MAPK signaling leads to increased phosphorylation and stabilization of TWIST1 [143], as well as increased transcription of TGF-β [144, 145]. Oncogenic mutations in the RAS/MAPK pathway, such as loss of NF1, a negative regulator of RAS, or activating mutations in BRAF and KRAS are common in cancer and have been shown to induce EMT [143, 146–151]. In these tumors, treatment with MEK inhibitors resulted in a reduction in EMT-associated proteins and even signs of induction of MET [145, 152–154]. In pediatric low-grade glioma (LGG), activating mutations of BRAF are common, and confer a worse prognosis [155]. In contrast, mutations in the RAS/MAPK pathway are rare in high-grade glioma, although loss of NF1 is sometimes found [136]. Yet, activation of the MAPK pathway is common, because of amplifications or mutations in EGFR and other RTKs [156]. In pediatric HGG, mutations in the RAS/MAPK pathway and loss of NF1 are even less frequent, compared to the adult disease with the lowest prevalence found in DIPG [138–140, 157]. Additionally, activating EGFR mutations are not found in pediatric HGG, although amplification of the EGFR and PDGFRA genes has been described [38, 49, 50, 139]. The significance of these findings in pediatric HGG is unclear; trials with MEK inhibitors in pediatric HGG and LGG give promising results [158], whereas a trial using the multi-kinase inhibitor sorafenib in pediatric low-grade glioma patients resulted in paradoxical progression of BRAF wild type tumors [159].

### The TAM family of receptor tyrosine kinases

Among the receptor tyrosine kinases, the TAM family, consisting of TYRO3, AXL and MERTK, plays a particular role in the induction of EMT. TYRO3 and MERTK can both be activated by binding of their endogenous ligands protein S and Gas6, whereas AXL can only bind Gas6 [160]. Additionally, AXL can form heterodimers with other RTKs, such as the EGF and PDGF receptors, thereby being transactivated by binding of their respective ligands [161]. Activation of TAM receptors triggers intracellular signaling cascades that have resulted in EMT in a large number of cancer types. Consequently, TAM signaling has been proven to be involved in migration of cancer cells in hepatocellular, colorectal and breast cancer, and resistance to chemo- and targeted therapy in breast cancer [161–173].

In glioblastoma, both AXL and MERTK have been identified as potential therapeutic targets and causes of treatment resistance. Inhibition of AXL and/or MERTK leads to diminished proliferation, migration and invasion of glioma cells, as well as sensitization of glioma cells to chemotherapy [174–180] Although specific studies addressing the biological role of the TAM RTKs in pediatric HGG and DIPG are not available, overexpression of both AXL and MERTK has been described, and AXL has been shown to be activated in cultured DIPG cells [180, 181].

### The JAK/STAT pathway in the mesenchymal transition

Besides the PI_3_K/AKT and RAS/MAPK pathway, a third pathway can be activated after engagement of cell surface receptors by their ligands; the JAK/STAT pathway. Although commonly associated with GPCRs, RTKs are also capable of initiating signaling along the JAK/STAT pathway. In this pathway, receptor-associated Janus kinases (JAKs) phosphorylate and activate signal transducer and activator of transcription (STAT) proteins. Upon phosphorylation, STATs form homo- or heterodimers that can function as transcription factors [182]. Within this family of proteins, STAT3 is known to induce EMT through upregulating the transcription of *SNAIL1*, *ZEB1*, *HIF*-*1α*, and most importantly, *TWIST* [183–187]. Under normal circumstances, activity of the JAK/STAT3/TWIST pathway is involved in neurogenesis and astrogliogenesis [188]. In cancer, however, aberrant activation of the pathway can induce EMT and thereby chemoresistance, as has been described, for example, in colorectal [184], breast [183] and ovarian cancer [189]. Based on these findings, clinical trials utilizing STAT3 inhibitors are currently ongoing for breast cancer [190, 191], ovarian cancer [189] and bile duct cancer [192]. In adult glioma, STAT3 activation is associated with increased tumor growth, angiogenesis, therapy resistance and worse patient outcome [12]. STAT3 activation enhances glioma stem cell self-renewal, as well as invasion and migration of glioma cells. Furthermore, inhibition of STAT3 enhances the response of glioma cells to temozolomide and radiation [193–196]. This suggests that inhibiting the JAK/STAT3 pathway may be an effective therapeutic strategy for high-grade glioma. The same might be true for pediatric HGG and DIPG, as overexpression of STAT3 has been reported as one of the hallmarks of the mesenchymal subtype in these tumors [38]. Additionally, overexpression of interleukin-4 and -13 receptors, known to induce JAK/STAT signaling, has been described in pediatric high-grade gliomas and DIPG [197–200].

### The sonic hedgehog pathway in the mesenchymal transition

Sonic hedgehog (SHH) is a soluble glycoprotein that can bind to the transmembrane receptor Patched1 (PTCH1), thereby inducing phosphorylation and stabilization of another transmembrane receptor, Smoothened (SMO). Activated SMO phosphorylates a variety of intracellular signaling molecules that eventually lead to increased activity of the GLI family of transcription factors [201, 202]. The GLI proteins, in turn, induce the expression of genes associated with EMT, such as *SNAI1*, *SNAI2*, *ZEB1*, *ZEB2* and *TWIST* [203, 204]. In the signaling cascade following activation of Smoothened there is a large amount of crosstalk with other pathways involved in EMT, such as the NOTCH, WNT/β-catenin, TGF-β and EGFR pathways [205, 206]. Aberrant activation of the Hedgehog pathway is involved in EMT in various malignancies, such as breast cancer and gastric cancer [207, 208]. In high-grade glioma cells, it has been shown to be involved in invasion, migration, proliferation and maintenance of stem cell properties [205, 209]. Moreover, high activity of the hedgehog pathway is correlated to worse outcome in patients suffering from HGG [210].

Inhibition of SMO represents a promising strategy to overcome therapy resistance in HGG; the Smoothened inhibitor cyclopamine has been shown to induce radiosensitization in glioblastoma cells and Erismodegib, another SMO inhibitor, was capable of inhibiting self-renewal and EMT in glioblastoma initiating cells [211, 212]. Additionally, chemical inhibition of the hedgehog pathway could sensitize glioma stem cells to temozolomide [213].

The hedgehog pathway has been found to be active in DIPG cells, where it plays a crucial role in self-renewal of malignant cells [214]. Its connection with the mesenchymal transition described in DIPG has not been studied yet.

### The NOTCH pathway in the mesenchymal transition

**Fig. 3:**
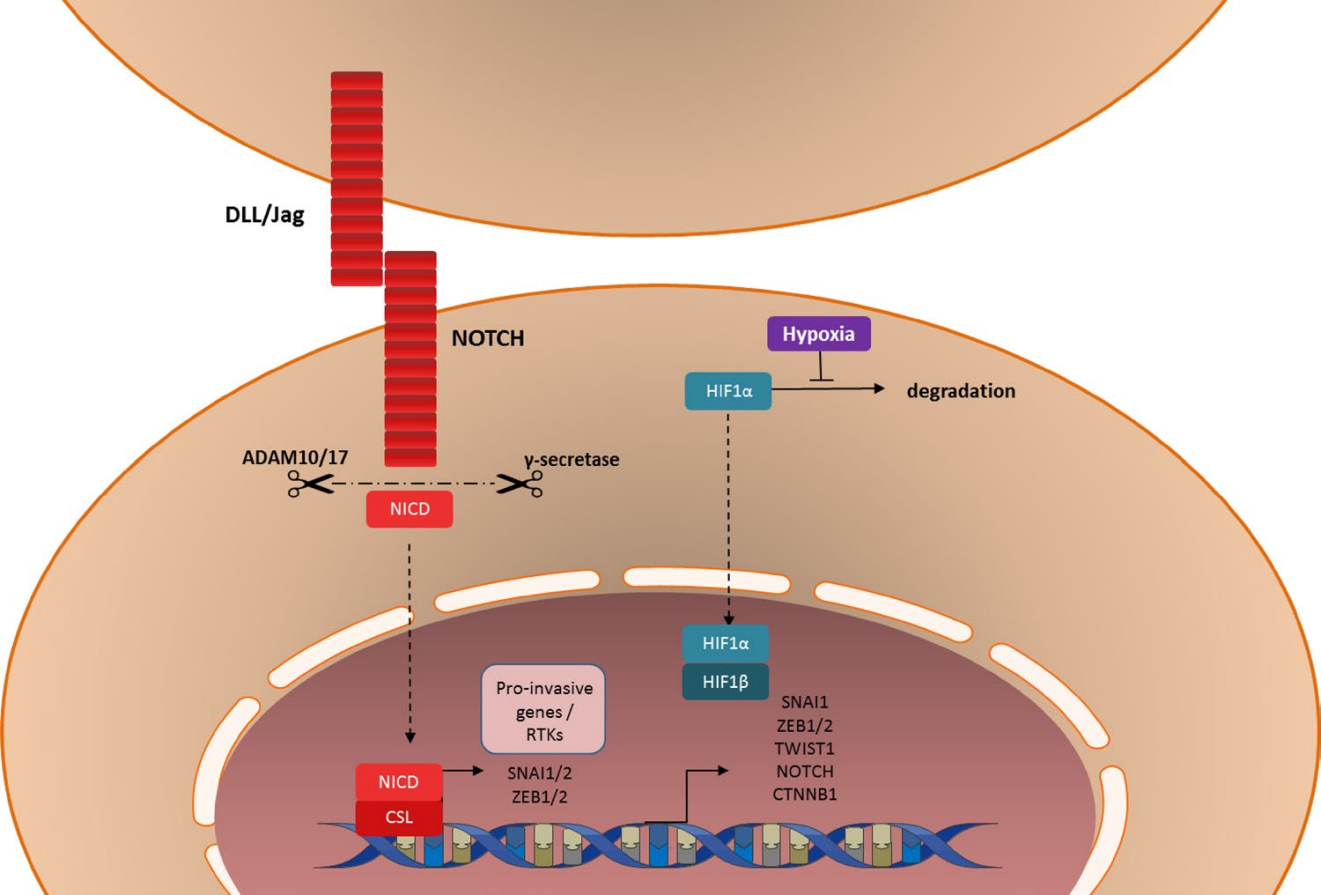
Graphic illustration of NOTCH signaling and hypoxia in the induction of the (epithelial-to-)mesenchymal transition

Mammalian cells express four transmembrane NOTCH receptors, NOTCH 1–4, and five canonical transmembrane ligands: Delta-like 1, Delta- like 3, Delta- like 4, Jagged-1 and Jagged-2. [215] When a NOTCH ligand binds to two adjacent NOTCH receptors of neighboring cells, NOTCH will be cleaved into an extracellular and an intracellular domain, by a dysintegrin and metalloproteinase domain-containing protein (ADAM) 10 or ADAM 17 and ƴ-secretase (Fig. 3). The active intracellular domain of NOTCH translocates to the nucleus, binds to C-protein binding factor 1/Suppressor of Hairless/Lag-1 (CSL), forming a transcription activator complex. The NOTCH-CSL complex induces transcription of numerous genes associated with differentiation and/or survival including a variety of genes associated with EMT, such as *SNAIL1*, *SNAIL2*, *ZEB1*, *FN1* and *PDGFR*s [216–218]. Furthermore, there is evidence of extensive crosstalk between the TGF-β and NOTCH pathways in the process of EMT [219, 220]. As with many other factors involved in EMT, the normal function of the NOTCH pathway seems to be in embryonic development and stem cell maintenance [219]. However, the NOTCH pathway is commonly dysregulated in cancer cells, where it can serve as both a tumor suppressor and as an oncogene, dependent on the cancer type [219]. NOTCH signaling induces proliferation, migration and invasion of glioma cells and is required for maintenance of both normal neural stem cells and glioma stem cells [221–224]. In these stem cells, NOTCH signaling induces self-renewal and inhibits differentiation [217, 225, 226]. Consequently, high levels of NOTCH signaling are associated with a poor prognosis in patients suffering from GBM [224]. γ-Secretase inhibitors have been shown to reduce proliferation and signs of stemness in glioma cells and to sensitize them to chemotherapy and radiation [227–231]. Moreover, these inhibitors have been shown to reduce proliferation of DIPG cells in vitro and to increase their radiosensitivity [232]. Although the role of NOTCH in EMT in pediatric HGG and DIPG has not been addressed yet, these results make it an interesting target for future translational research.

### Role of hypoxia in the mesenchymal transition

Another external factor, other than growth factors and cytokines that is capable of inducing mesenchymal transition is hypoxia. Exposure of tumor cells to hypoxia induces stabilization of hypoxia-inducible factor 1α (HIF-1α), which dimerizes with HIF-1β to form a transcription factor complex (Fig. 3). Activity of this complex results in expression of genes involved in EMT, including *NOTCH*, *CTNNB1* (β-catenin), *SNAIL1* and *TWIST* [233–239]. Additionally, the expression of HIF-2α (EPAS1) is associated with ZEB2-induced EMT in breast cancer [240].

Besides EMT, these hypoxia-inducible factors induce expression of genes responsible for stem cell properties, such as self-renewal and multipotency via mechanisms common to EMT, such as the NOTCH signaling pathway [241]. Furthermore, in many different cancer types, hypoxia-inducible factors are involved in proliferation, migration and invasion, reinforcing the hypothesis that hypoxia can initiate EMT in cancer cells [235, 237–239, 242, 243]. In glioblastoma cells specifically, hypoxia induces and maintains stem cell characteristics, and HIF-1α has been shown to be involved in proliferation, migration invasion and radio- and chemoresistance [238, 239, 244–252]. In both adult and pediatric HGG, overexpression of HIF-2α has been found, which negatively correlated with patient survival [247, 253], although data on HIF-1α in pediatric HGG is lacking.

## The cadherin switch

The ultimate result of activation of the EMT-related signaling pathways described above is the so-called cadherin switch [254–257] (Fig. 1). Cadherins are a large family of transmembrane glycoproteins involved in intercellular adhesions and signaling. Epithelial cells normally express E-cadherin, the expression of which is tightly regulated. The SNAIL and ZEB families of transcription factors bind to an E-Box motif in the promoter region of the CDH1 gene, causing its transcriptional repression [11, 23, 41]. Simultaneously, they induce an upregulation of other cadherins, mostly CDH2 and/or CDH4 [11]. Other transcription factors involved in the mesenchymal transition, such as TCF3 (E12/E47), and the TWIST and SIX families of transcription factors, induce similar changes in cadherin expression via different mechanisms than described above [11, 254–257]. Loss of expression of E-cadherin (CDH1), and induction of expression of N- or R-cadherin (CDH2 or CDH4), triggers a series of events that allows cancer cells to become less dependent of their micro-environment, thereby promoting cell migration, invasion and metastasis [254–257]. Additionally, the intracellular domain of E-cadherin binds β-catenin(CTNNB1), and loss of its expression causes β-catenin to locate to the nucleus where it acts as a transcription factor for various genes associated with the mesenchymal phenotype [257, 258]. The broad range of functions attributed to cadherins also includes profound influences on several signal transduction pathways, such as the PI_3_K/AKT, MAPK and NF-kB pathways, both directly and through modulation of receptor tyrosine kinases such as FGFR, EGFR, PDGFR and integrin-linked kinases [259–263].

In adult glioblastoma (GBM), loss of E-cadherin expression and concurrent increase in either N- or R-cadherin expression is related to a more mesenchymal phenotype, tumor invasiveness and worse overall survival [254, 260, 264–266]. Furthermore, the mesenchymal phenotype seems to be responsible for an increased resistance to chemotherapy and radiation, two important modalities in GBM treatment [15]. To this date, the role of cadherins in pediatric high-grade glioma has not been sufficiently addressed to determine their precise role in the mesenchymal transition in these tumors.

## Discussion

Over the past years, the mesenchymal transition has received considerable attention in the field of oncology, due to its relationship with invasion, metastasis and therapy resistance of cancer cells. Components of the mesenchymal transition have often been put forward as therapeutic targets and/or therapy-sensitizing agents, not in the least in the treatment of adult glioblastoma. However, little research is available on the role of the mesenchymal transition in pediatric high-grade glioma and diffuse intrinsic pontine glioma. The therapy resistance and diffusely infiltrative nature that these tumors often possess imply that mesenchymal gene expression may play a larger role in their biology than is known so far. We have reviewed current knowledge about the major signal transduction pathways involved in initiation and maintenance of the mesenchymal transition in cancer in general, and high-grade glioma in particular.

**Fig. 4:**
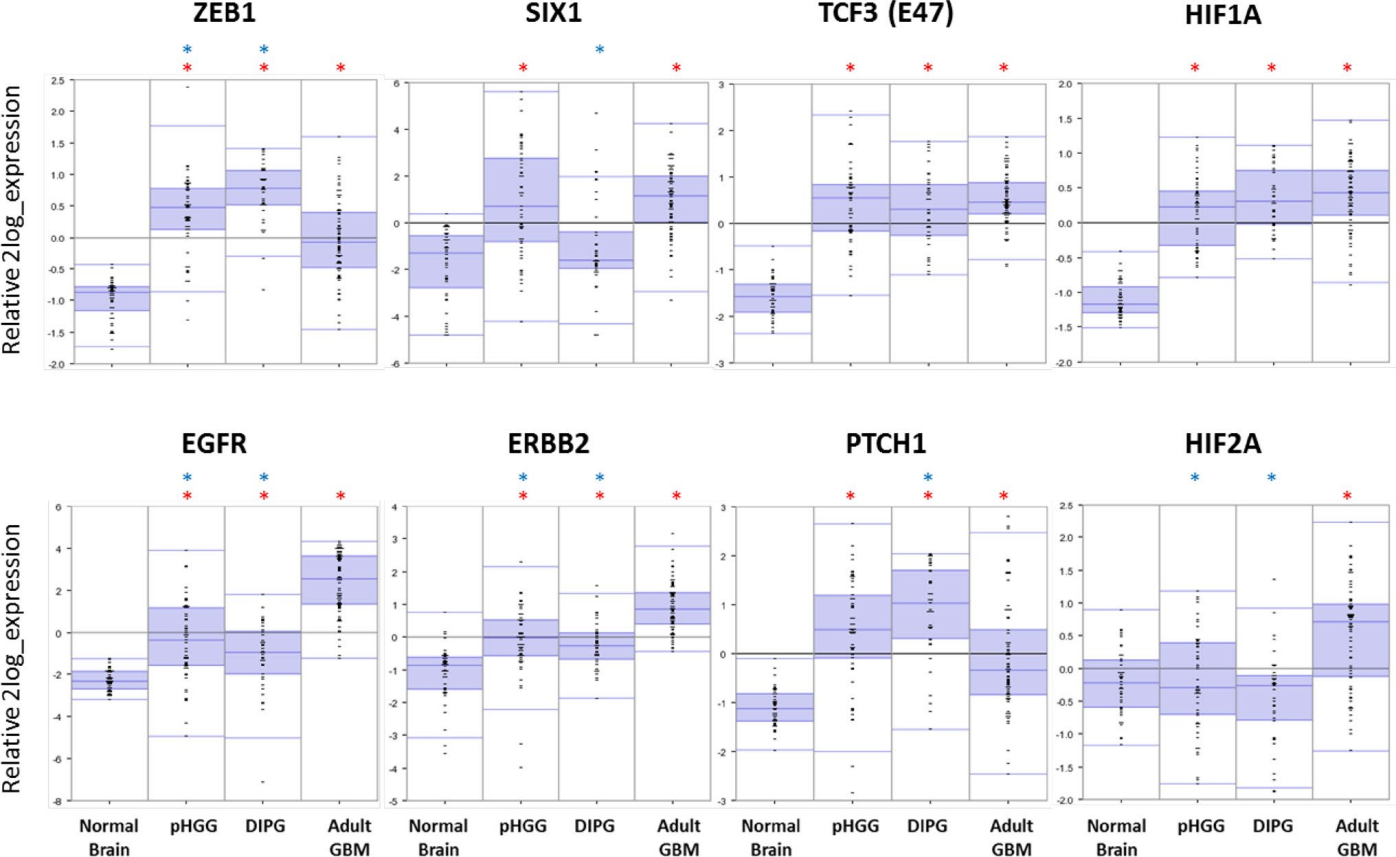
RNA expression levels of genes involved in the (epithelial-to-)mesenchymal transition that show a strong differential expression between normal brain tissue [267], adult GBM [268], pediatric HGG [49] and DIPG [48]. Source: R2 (http://r2.amc.nl). Blue stars indicate significant differences (*p* < 0.01) compared to adult GBM, red stars indicate significant differences (*p* < 0.01) compared to normal brain tissue

When comparing published RNA expression datasets available from normal brain tissue [267], adult GBM [268], pediatric HGG [49] and DIPG [48], major differences can be found in the expression of certain key components of the mesenchymal transition (Fig. 4). Most prominently, expression of the transcription factor ZEB1 is higher in pHGG and DIPG compared to adult GBM, whereas expression of SIX1 is upregulated in both pHGG and adult GBM, but not in DIPG. This is in contrast with other transcription factors, such as TCF3, the expression of which was equal in all three tumor types. Expression of upstream regulators of transcription factors involved in the mesenchymal transition revealed further differences between pHGG, DIPG and adult GBM. Most strikingly, the expression of EGFR and ERBB2—two commonly targeted RTKs in the treatment of glioma—was much higher in adult GBM compared to pHGG and DIPG. Combined with the observation from previous studies that EGFR mutations are rarely to never found in pediatric HGG and DIPG, these data suggest that the role of EGFR signaling in pediatric gliomas is more limited than in the adult counterpart [38, 49, 50, 139]. On the contrary, the expression of Patched (PTCH1), an important component of the hedgehog pathway, was higher in DIPG than in supratentorial pHGG and adult GBM, possibly as a result of their different developmental origins [214]. Finally, in contrast to previous reports describing overexpression of *HIF2A* in both pediatric and adult HGG [247, 253], only adult GBM possessed *HIF2A* overexpression in the datasets we analyzed, whereas *HIF1A* was overexpressed in all three tumor types. Tables 1 and 2 present a brief overview of transcription factors and signaling pathways addressed in this review and compare their relative importance in pHGG, DIPG and adult GBM, as far as current knowledge allows for.

**Table 1:** Summary of major transcription factors involved in the mesenchymal transition and their role in adult GBM, pediatric HGG and DIPG

Transcription factors	Relevance in adult GBM, pediatric HGG and DIPG
SNAIL family	Proven important role in mesenchymal transition in adult GBM, suggested to play a role in DIPG, insufficient data on pediatric HGG
ZEB family	Proven role in mesenchymal transition in adult GBM and pediatric HGG, insufficient data on DIPG. The presence of a PDGFRα-ZEB1 axis and expression levels of ZEB1/2 suggest important role in pediatric HGG and DIPG
TWIST family	Proven important role in mesenchymal transition in adult GBM, no available data on pediatric HGG and DIPG
SIX family	Proven role in mesenchymal transition in adult HGG, no available data on pediatric HGG and DIPG. Expression data suggest inferior role in DIPG compared to adult and pediatric supratentorial HGG

**Table 2:** Summary of major signaling pathways involved in the mesenchymal transition and their role in adult GBM, pediatric HGG and DIPG

Signaling pathway	Relevance in adult GBM, pediatric HGG and DIPG
TGF-β/activin	Proven role for classic TGF-β signaling in mesenchymal transition in adult GBM, insufficient data on pediatric HGG and DIPG. The presence of ACVR1 mutations in DIPG suggests important role for activin signaling in mesenchymal transition in these tumors
WNT/β-catenin	Proven role of canonical WNT signaling in mesenchymal transition in adult GBM, likely limited role in pediatric HGG, insufficient data on DIPG. Non-canonical WNT signaling seems to have a role in mesenchymal transition in adult GBM; no data is available on pediatric HGG and DIPG
PI_3_K/AKT	Strong suggestions for role in mesenchymal transition in adult GBM, pediatric HGG and DIPG. RTK overexpression and activation and PI_3_K mutations in pediatric HGG and DIPG support this role
RAS/MAPK	Suggestions for role of increased RAS/MAPK signaling in mesenchymal transition in adult GBM, insufficient data on pediatric HGG and DIPG. Mutations in BRAF and NF1 may have a role in some patients. Pathway activation and possible induction of mesenchymal transition is largely mediated by RTK overexpression and activation in these tumors
TAM RTK family	Proven role for AXL and MERTK in mesenchymal transition in adult GBM and pediatric HGG, no data on DIPG
JAK/STAT	Suggested role in mesenchymal transition in adult GBM, pediatric HGG and GBM. Possible involvement of overexpressed IL-4 and IL-13 receptors in JAK/STAT-mediated induction of mesenchymal transition in pHGG and DIPG
Sonic hedgehog	Strongly suggested role in mesenchymal transition in adult GBM. Proven important in initiation and progression of DIPG, although connection with mesenchymal transition not described. No data available on pediatric HGG with regard to mesenchymal transition
NOTCH	Suggested role in mesenchymal transition in adult GBM, insufficient data concerning pediatric HGG and DIPG
Hypoxia	Suggested role in mesenchymal transition in adult and pediatric HGG, data lacking on DIPG

The differences in mechanisms of mesenchymal transition employed by pHGG and DIPG as compared to adult GBM will provide researchers with important clues in the identification of therapeutic targets and the design of novel therapeutic strategies to overcome therapy resistance and metastasis in these hard-to-treat brain tumors. With the currently available knowledge, several promising therapeutic targets for interfering with the mesenchymal transition in pHGG and DIPG can already be identified. With the development of selective ACVR1 inhibitors, tumors harbouring activating *ACVR1* mutations and a mesenchymal phenotype can potentially be chemo- and radio-sensitized using these compounds [269]. Furthermore, tumors possessing a mesenchymal phenotype related to mutations in the PI_3_K and/or MAPK pathway can potentially be sensitized to therapy by the use of PI_3_K or MEK inhibitors, many of which are already clinically approved for other indications. Also, considering the perceived importance of the hedgehog pathway in the development and progression of DIPG, currently available Smoothened inhibitors may be used to interfere with the mesenchymal transition in this type of cancer. Within the paradigm of the mesenchymal transition, these drugs would only serve as therapy sensitizers, and should therefore always be combined with other cytotoxic therapeutics or radiotherapy. Finally, over the past years, it has become clear that a large part of pediatric HGG and DIPG are driven by mutations in genes encoding the Histone 3 protein. These mutations either induce a K27M or G34R/V substitution in the N-terminal end of the Histone 3 protein, thereby preventing histone methyltransferases from methylating H3K27 or H3K36 and causing a global epigenetic shift [3–7, 270]. This hypomethylation of Histone 3 at the K27 or K36 residues causes genome-wide changes in gene expression. Of these, the gene expression changes induced by the H3K27M mutation have been extensively investigated [270–273]. Despite this thorough research, the influence of the Histone 3 mutations on the expression of genes involved in the mesenchymal transition has not been specifically addressed yet. Given the observation that differences in expression of mesenchymal genes exist between DIPG carrying a H3.1 or H3.3 K27M mutation, it is not unlikely that the epigenetic shift induced by these mutations is capable of influencing or even inducing the mesenchymal transition in these tumors [38]. These preliminary data warrant further investigation of the connection between Histone 3 mutations, the resulting changes in histone methylation, and the mesenchymal transition in these tumors.
